# Copy or collaborate? How networks impact collective problem solving

**DOI:** 10.1038/s44260-025-00058-8

**Published:** 2025-11-27

**Authors:** Gülşah Akçakır, John C. Lang, P. J. Lamberson

**Affiliations:** 1 Department of Communication, UCLA, Los Angeles, CA USA; 2 Health Economic and Decision Sciences, Biostatistics and Research Decision Sciences, Merck Canada Inc., Merck, Canada

**Keywords:** Communication, Decision making

## Abstract

Collaboration enables groups to solve problems beyond the reach of their individual members in contexts ranging from research and development to high-energy physics. While communication networks play a pivotal role in group success, there is a longstanding debate on the optimal network topology for solving complex problems. Prior research reaches contradictory conclusions–some studies suggest networks that slow information transmission help maintain diversity, leading groups to *explore* more of the problem space and find better solutions in the long run, while others argue that networks that maximize communication efficiency allow groups to *exploit* known solutions, boosting overall performance. Many existing models assume that individuals use their network connections only to copy better-performing group members, but we show that such groups often perform worse than if individuals worked independently. Instead, our model introduces a crucial distinction: in addition to copying, individuals can actively *collaborate*, leveraging diverse perspectives to uncover solutions that would otherwise remain inaccessible. Our findings reveal that the optimal network structure depends on the balance between copying and collaboration. When copying dominates, inefficient, exploration-focused networks lead to better outcomes. However, when individuals primarily collaborate, highly connected, efficient networks win out. We also show how groups can reap the benefits of both strategies by employing a collaborate first-copy later heuristic in highly connected networks. The results offer new insights into how organizations should be structured to maximize problem-solving performance across different contexts.

## Introduction

In 1905, Einstein was stuck, flummoxed by contradictions in Newtonian mechanics and Maxwell’s equations of electromagnetism. He famously turned to his friend and fellow patent office employee, Michele Besso. During a long night of discussion, Besso reminded Einstein of a central idea promoted by the Austrian physicist Ernst Mach: all measurements are relative. The next morning, Einstein returned to his friend saying, “Thank you, I’ve completely solved the problem.” Shortly thereafter, he published the first paper on his theory of special relativity, arguably one of the most significant scientific papers in history, with Besso as the sole acknowledgement^1^.

In 1953, while racing to develop a model of DNA’s structure, the molecular biologist James Watson visited King’s College London. There, he saw Rosalind Franklin’s now famous Photograph 51. With her expertise in chemistry and X-ray crystallography, Franklin created the image using a special system she developed that involved bubbling hydrogen through salt solutions, allowing her to capture a much clearer image of the high humidity “B-form” of the nucleic acid than ever seen before^2^. In Watson’s words, “The instant I saw the picture my mouth fell open and my pulse began to race”^3^. The unique perspective gained from Franklin’s image allowed Watson and Crick to complete the model that ultimately led to a Nobel Prize in 1962.

In 2009, Fields medalist Timothy Gowers challenged his blog’s readers to find a combinatorial proof of the density Hales-Jewitt Theorem–a problem he himself had been unable to solve^4^. After six weeks and nearly 1000 comments from individuals with experience ranging from a fellow Fields medalist to high school mathematics teachers, Gowers declared the team of online collaborators had collectively found the proof, which was published under the pseudonym D.H.J. Polymath ^5,6^.

These three examples are among countless instances in which, by combining diverse perspectives and expertise, groups were able to solve complex problems collectively that none of their individual members could overcome alone^7,8^. Research on the emergence of such collective intelligence points to the connections among collaborators–that is, the group network structure–as a key ingredient in problem-solving solving success^9,10^. And yet, the literature abounds with contradictory conclusions on which networks work best. On the one hand, a number of experimental studies find that highly connected networks with short average path lengths efficiently diffuse information, promote coordination, and allow groups to rapidly converge on and *exploit* discovered solutions^11–13^. On the other hand, a collection of theoretical models suggests that less efficient networks should help organizations maintain diversity and *explore* a greater range of possible solutions ^14–16^.

We argue that one reason for discrepancies between theory and empirics regarding optimal networks develops because most models of networked problem solving focus on a limited type of communication and learning among network neighbors: copying^14,15,17^. Even in more sophisticated social learning models, individuals ultimately mimic the solutions found by more successful group members^18^. A perhaps unintended consequence of the copying assumption in previous research is that a group never outperforms the same team members working as individuals. In other words, there is no “synergy” and no possibility of a collective breakthrough like those that led to the theory of special relativity or the discovery of the structure of DNA. In this paper, we relax the copying assumption by endowing agents with diverse problem-solving perspectives, which they share through a second form of communication we term *collaboration*. As in real-world problem-solving groups, when agents in our model collaborate, they have the potential to jointly outperform all of the individuals in the group. We then study how the nature of information sharing interacts with network structure and problem complexity to shape the exploration/exploitation tradeoff and determine organizational performance.

Our article makes three main contributions. First, we demonstrate how group performance depends on the extent to which group members use their network connections for copying versus collaboration and find a tradeoff between average and best group member performance. Second, we resolve the apparent conflict in the literature on the relationship between networks and problem solving by demonstrating that when agents are restricted to copying, inefficient networks that slow communication are most effective, but when agents use their networks primarily for collaboration, more efficient networks are best. Finally, we show how an organization can further optimize performance by changing its primary communication mode over time.

**Fig. 1:**
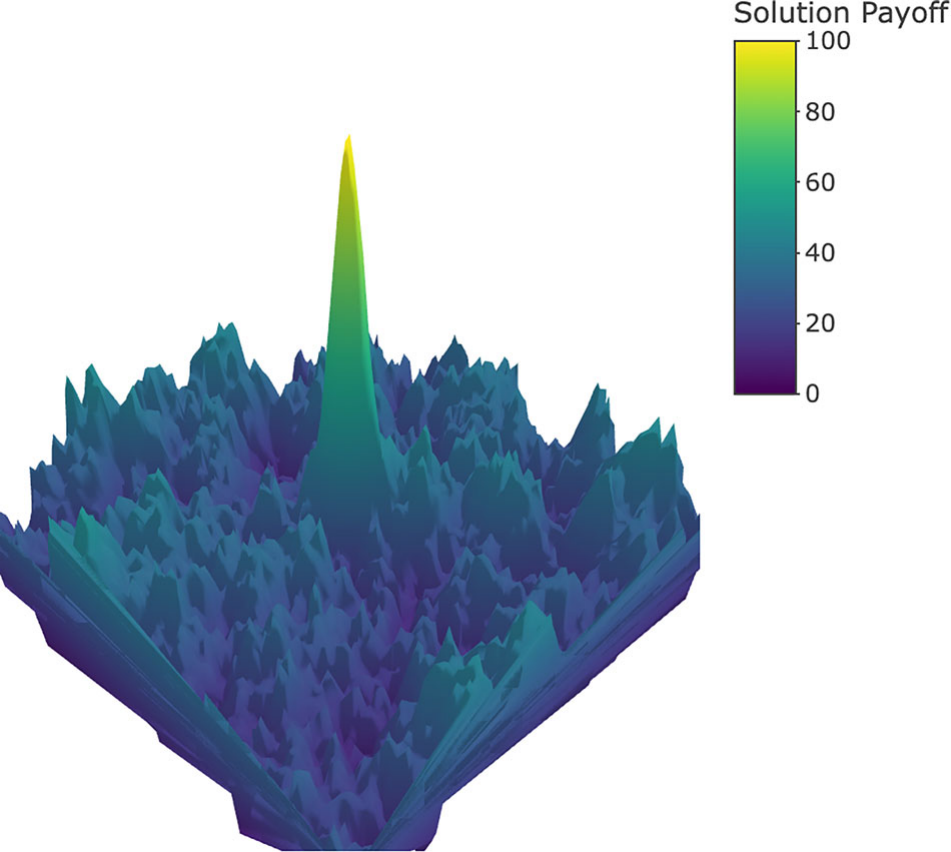
An example of the rugged solution landscape. The two horizontal dimensions specify characteristics of each potential problem solution. The vertical axis indicates each solution’s payoff.

Following previous studies, we model problem solving by simulating agents searching for the global maximum on a rugged landscape^13,14,17–19^. As shown in Fig. 1, solutions are represented as points on a surface embedded in three-dimensional space, with their height representing their payoff. (In the Supplementary Information (SI) we show that our results also hold when we replace this two-dimensional surface with a multidimensional NK-landscape^20^.) Each agent occupies a point on the landscape corresponding to that agent’s current solution. Agents are only aware of the payoffs of solutions in the local neighborhood of their current solution and attempt to find the highest peak, or global optimum, by “hill climbing,” that is, repeatedly moving to the highest payoff solution that they can “see”^21^. As an agent improves upon its current solution, new possibilities come into view. The presence of many local optima complicates the search for the globally optimal solution because hill-climbing agents may become stuck at the top of these local peaks with no further improvements in sight.

**Fig. 2:**
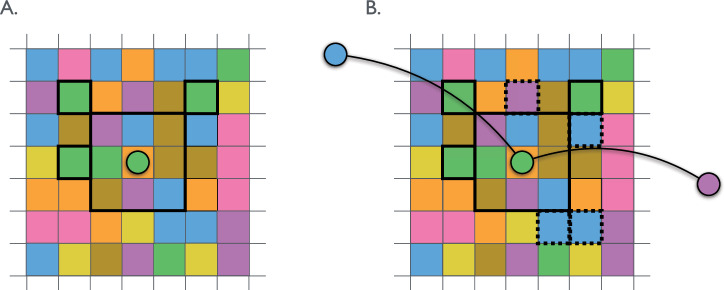
A local neighborhood in the problem space grid. Colors corresponds to the skill type of each cell. **A** An agent represented by the green circle in the center can see the payoffs of any of the cells surrounded by bold lines. This includes the central orange cell they currently occupy, the eight immediately adjacent cells, and three additional cells that match the agent’s green skill type and are within distance $r=2\sqrt{2}$. **B** If the agent collaborates with their network neighbors, represented by the blue and purple circles, then they also can see the payoffs of the blue and purple cells with dashed borders.

To extend our model beyond this standard setup, we draw from the “wisdom of crowds” literature^7^, which argues that groups working together combine diverse perspectives, allowing them to often outperform their individual members ^8,17,22–24^. We operationalize perspective diversity by assuming that the set of solutions visible from a given point on the solution landscape differs from agent to agent. We divide the two horizontal dimensions of the problem-solving environment into a grid, where each cell represents a possible problem solution. As described above, individuals are aware of the payoffs of cells immediately adjacent to their current solution, but on top of this standard framework, each cell and each agent is randomly matched with one of *s* possible “skills”. In addition to the adjacent cells, agents know the payoffs of, and can move to, cells that match their skill up to a distance *r* from their current position. The left panel of Fig. 2 illustrates the framework with colors representing skills and $r=2\sqrt{2}$. The green skill agent in the center can see all of the cells outlined in bold, which includes the orange cell they currently occupy, the eight immediately adjacent cells, and three additional cells one step further away that match their green skill type.

The applied interpretation of skills depends on context. In the case of academic collaborations, skills might correspond to disciplines, while in the medical arena, skills could represent subfields such as cardiology, oncology, or endocrinology. Skills could also correspond to individual knowledge gained through idiosyncratic past experiences. While any agent can potentially reach any solution, it is easier for an individual to “make the leap” to a solution that matches their type. For example, given equivalent information on a patient, a cardiologist might more easily pick up on signs of a heart problem than a gastroenterologist.

Agents are connected by an unweighted, undirected social network. In addition to individual local knowledge of the problem space, agents gain information through their network connections. When an individual communicates with a network neighbor, they can either *copy* or *collaborate*, and we vary the frequency of these two options, letting *p* represent the rate of collaboration. Specifically, when it is an agent’s turn to update, with probability *p* they collaborate with their neighbors and with probability 1 − *p* they copy.

As in previous models, when an agent copies, the individual moves to the position of their best performing network neighbor if the payoff of the cell occupied by that neighbor exceeds the payoff of any currently visible solutions in the agent’s local neighborhood^14,15,25^. If none of an agent’s neighbors’ solutions exceed the payoffs in the agent’s visible neighborhood, they follow the hill-climbing algorithm and adopt the solution with the highest payoff in their visible neighborhood. When collaborating, the agent learns the payoffs of cells within *r* units of their own position that match the skills of their neighbors. Just as Einstein turned to Besso for a new perspective, when agents in the model collaborate, it is as though they ask their neighbors, “Given your skills, if you were in my position, what steps forward do you see?” The collaborator essentially responds, “Have you thought of this?” The knowledge gained by collaborating is illustrated in the right panel of Fig. 2 by the cells with dashed borders. On their own, the green skill agent can only see the cells within the dark border shown on the left side of the figure. But when they collaborate, they learn the values of the blue and pink cells with dashed borders on the right side of the figure through their interaction with their blue and pink skill neighbors. Once the agent learns the values of these additional solutions, they move to the cell with the highest payoffs out of all of the cells they know.

We also vary the structure of the collaboration network. Since network efficiency, as measured by the average path length between any two network nodes, has been identified as a primary predictor of group performance^10^, we focus on two extremes: a fully connected network, in which every individual is directly linked to everyone else in the group; and a linear network, in which agents are arranged in a line and are only connected to their immediate neighbors. These two networks have the shortest and longest possible average path lengths, respectively, among all connected graphs. In addition to these extremes, we consider eight network structures examined in previous studies of team networks^13,18^ in the SI.

## Results

### Diverse skills and the wisdom of crowds

**Fig. 3:**
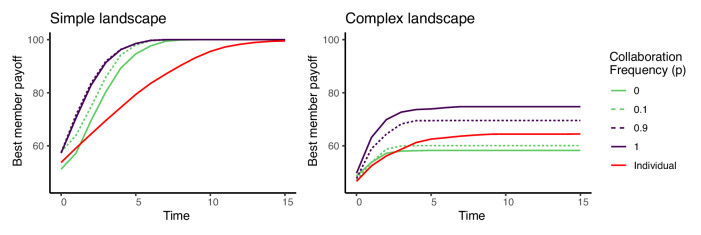
The best solution found by any member in the group over time for varying levels of collaboration frequency. Averages from one thousand simulation runs with group size *n* = 16, *s* = 100 skill types, and skill radius *r* = 6. Simulations terminate after a single time step in which no agent moves.

Before investigating variations in network structure, we examine the impact of collaboration on collective problem solving. Figure 3 shows the maximum payoffs achieved over time when agents only or mostly copy (*p* = 0 and *p* = 0. 1, respectively) or only or mostly collaborate (*p* = 1 and *p* = 0. 9, respectively), along with a baseline case where agents work as individuals, sharing no information with other group members. The left panel illustrates the findings in a simple problem landscape with a single global optimum and no other local optima, while the right panel shows the results in a complex landscape with many local optima, like the one depicted in Fig. 1. Agents communicate through a fully connected network in all cases. In the simple landscape, there is little difference in performance between the different communication profiles, although all of the strategies outperform a group working as individuals.

Results from the complex landscape shown on the right reveal a different dynamic. The increased complexity of the problem space reduces group performance across all conditions relative to the simple landscape, but now agents that collaborate frequently find much higher payoff solutions than those who primarily copy. Comparing with agents that search individually reveals two reasons for the advantage of collaboration over copying. First, copying is actually detrimental to group performance relative to individual search. Because copying reduces the set of solutions explored by the group relative to independent searching, it lowers the chance of a group member locating the global maximum. In terms of the best solution identified by any member of the group, teams from some previous models of collective problem solving would have been better off working as individuals than sharing information ^14^^,^^18^. Part of the reason collaboration outperforms copying is simply because more frequent collaboration implies less copying. However, collaboration also offers additional benefits beyond the reduction of copying, as shown by the increase in performance of the all collaboration (*p* = 1) and mostly collaborate (*p* = . 9) strategies relative to the group working as individuals. By combining their diverse skills, agents who collaborate are less likely to get stuck on local optima, and thus they find higher payoff solutions overall.

**Fig. 4:**
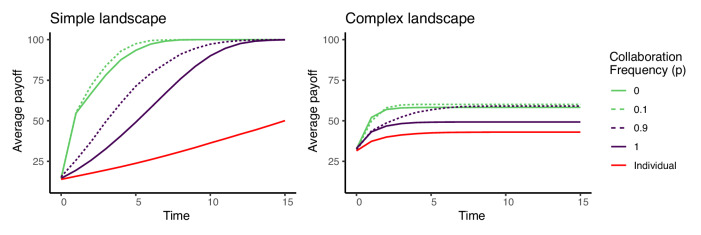
The average group performance over time for varying levels of collaboration frequency. Averages from one thousand simulation runs with group size *n* = 16, *s* = 100 skill types, and skill radius *r* = 6. Simulations terminate after a single time step in which no agent moves.

These results beg the question, why should group members ever copy? Fig. 4, which shows the average group performance over time for the same five communication profiles as the previous figure, provides one answer. While copying lowers the best payoff solution found by the group, it raises the group’s average member performance in both simple and complex problem landscapes. Collaboration allows a group to explore a larger area of the problem landscape, raising the payoff of the best performing agent, but when other group members do not have an opportunity to copy one another, the rest of the group is unable to exploit the success of the best performing member. Whether average group performance or best member performance matters more depends on context. A team of engineers working to solve an R&D design challenge may only need one member to find a breakthrough solution, but for a group of bees gathering food for the hive, it matters little for one bee to find the mother lode if the other workers come home empty. Notably, only a small proportion of copying is needed to close the gap in average performance between the all collaboration strategy and the primarily copying strategies in the long run. If agents copy at all, eventually the entire group will converge on the best member’s solution, although it may take longer.

### Interaction of network structure and communication type

As shown in the previous section, the introduction of diverse skills and collaboration allows groups to collectively outperform their individual members. Prior research identifies network structure as another key determinant of group problem-solving performance^10,13–15,18,25–28^. In this section, we examine how communication type and network structure interact by varying the frequency with which agents collaborate with or copy their network neighbors, as well as the network that connects them.

As discussed above, several papers reach opposing conclusions regarding the optimal network structure for group performance. For example, using an agent-based computational model, Lazer and Friedman conclude that “the more efficient the network at disseminating information, …the lower the long-run performance of the system” ^14^. In contrast, in an experimental setting, Mason and Watts find that “efficient networks perform unambiguously better than inefficient networks”^13^. Barkoczi and Galesic show that differences in how individual agents determine which of their neighbors to copy provide one possible explanation for this contradiction^18^. As we demonstrate below, communication content can also resolve this inconsistency.

**Fig. 5:**
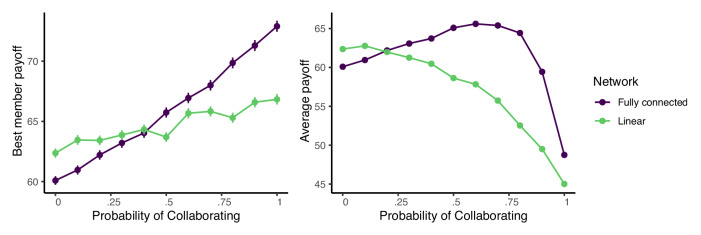
The maximum (left) and average (right) group payoffs in fully connected and linear networks as a function of the probability of collaboration, *p*. Error bars are the standard errors of the mean across one thousand simulation runs. Parameters: group size *n* = 16, *s* = 100 skill types, and skill radius *r* = 6. Simulations terminate after a single time step in which no agent moves.

Figure 5 shows the best member (left) and average (right) payoffs when agents are connected by either a linear network (green) or a fully connected network (purple) as a function of the collaboration frequency, *p*. When the probability of collaboration is zero the results are consistent with Lazer and Friedman’s model, and the inefficient linear network outperforms the maximally efficient fully connected network^14^.

At the other extreme, when *p* = 1, agents always collaborate and the opposite result obtains: the fully connected network outperforms the linear network in terms of both best member and average payoffs, replicating the finding from Mason and Watts^13^. Between the two extremes, the performance curves for the two networks cross: the linear network performs best when agents primarily copy, while the fully connected network dominates when agents collaborate more frequently.

Figure 5 provides another illustration of the tradeoff between the best member and average performance that comes with increased collaboration. On the left, greater collaboration boosts the best member performance in both the linear and fully connected networks, but as the right panel depicts, too much collaboration reduces the payoff of the average group member by preventing good solutions from diffusing throughout the network.

**Fig. 6:**
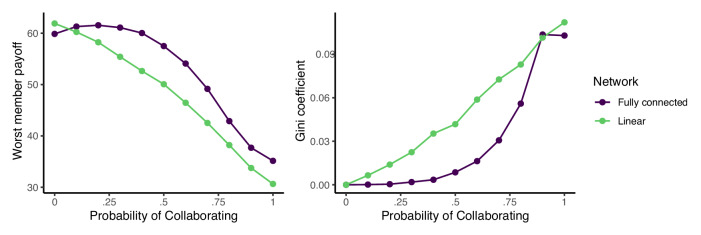
The minimum (left) group payoffs and Gini coefficient (right) in fully connected and linear networks as a function of the probability of collaboration, *p*. Error bars are the standard errors of the mean across one thousand simulation runs. Parameters: group size *n* = 16, *s* = 100 skill types, and skill radius *r* = 6. Simulations terminate after a single time step in which no agent moves.

The results in Fig. 5 suggest that too much collaboration increases inequality: while some team members surge ahead, the average member is left behind. This intuition is confirmed in Fig. 6, which plots the payoff of the worst performing team member in the left panel and the Gini coefficient in the right panel for the same simulations depicted in Fig. 5. While the performance of the best team member improves with increased collaboration (Fig. 5), the performance of the worst team member drops, and overall inequality climbs. Thus, in situations where fairness or equality are important considerations, higher levels of copying may be preferable.

**Fig. 7:**
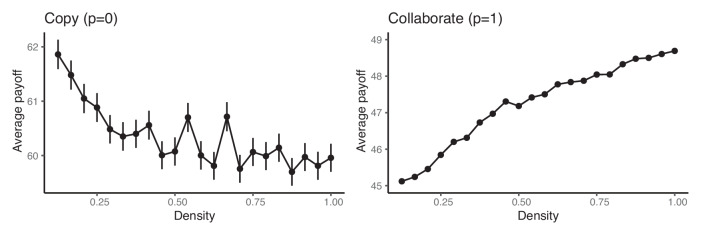
The relationship between average group performance and network density when agents only collaborate (left) or only copy (right). The density of the network is the number of edges in the network divided by the number of edges there would be in a fully connected network with the same set of nodes. Network density was increased by starting with a linear network and adding edges at random. Error bars are the standard errors of the mean across one thousand simulation runs. Parameters: group size *n* = 16, *s* = 100 skill types, and skill radius *r* = 6. Simulations terminate after a single time step in which no agent moves.

To this point, we have only examined two extreme networks: the linear network and the fully connected network (a number of other network structures are examined in the SI). Figure 7 expands on these results by depicting the average group performance as edges are added to the interaction network at random, increasing the density of network connections from the minimally connected linear network (density = 0.125) to a fully connected network (density = 1). When agents only copy, as shown in the left panel, increasing density decreases performance. On the other hand, as depicted in the right panel, when agents always collaborate, greater connectivity leads to higher payoffs. There is no “one size fits all” prescription on how organizations should best structure their problem-solving networks. Rather, the costs and benefits of increased communication depend on the information communicated.

### Collaborate first, then copy

The results above demonstrate advantages to both collaboration and copying. Collaboration amplifies exploration and increases the expected payoff of the best found solution while copying boosts exploitation, raising the payoff of the average group member. Typically, exploration and exploitation are seen in opposition to one another, as above, where the best strategy for the team’s maximum payoff was the worst for the group average. But, in this section, we explore a third heuristic, where members first collaborate and then later copy, which allows groups to capture the benefits of both strategies.

**Fig. 8:**
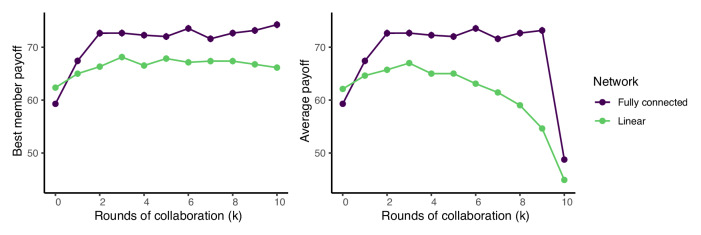
Average and maximum payoffs when groups only collaborate for *k* rounds and then only copy for 10 − k rounds as a function of how many rounds the group spends collaborating (*k*). Parameters: group size *n* = 16, *s* = 100 skill types, and skill radius *r* = 6.

Figure 8 shows the maximum and average payoffs when groups first collaborate for *k* rounds and then copy for 10 − *k* rounds as a function of *k*. When *k* = 0, agents always copy, and so the results mirror the *p* = 0 results on the left side of Fig. 5. If the group collaborates for at least one round, the fully connected network outperforms the linear network with respect to both best member and average payoffs. Because in the fully connected network it only takes one round of copying for all of the group members to match the performance of the best member, best member and average performance are the same. In these cases, the group enjoys the exploration benefits of collaborating in a fully connected network without paying the cost of premature conformity due to early copying. By copying later, the group also reaps the exploitation benefits that come from bringing the performance of the average group member up to the level of the best member. Now, having a fully connected network is actually beneficial for copying, because the group has already exhausted its ability to explore the space and the efficient network allows the whole group to quickly adapt the best found solution.

In the linear network, it takes longer for good solutions to diffuse throughout the network, so if the group collaborates for too long, there are insufficient rounds remaining for the best solutions to reach all of the group members. This accounts for the drop off in average performance in the linear network seen in the right panel. Finally, on the far right side of the right panel where *k* = 10, so the groups only collaborate, there is a sudden decrease in average performance because poor performing group members have no opportunity to learn the solutions found by better performing teammates.

## Discussion

We developed a model of networked problem solving in which, in addition to copying better-performing teammates, group members can also collaborate by sharing diverse skills with their network neighbors, resulting in groups that collectively outperform their individual members. We then explored how group performance depends on the content of group member communications, the network that structures group communication, and the interaction of the two. While copying tends to boost a group’s average payoff, it reduces the expected performance of the best solution found by the group. Collaboration increases the chance that someone in the group finds an outstanding solution, but too much collaboration comes at the cost of a decrease in the payoff of the average group member.

The model resolves contradictory results in the literature regarding network efficiency and group performance. Specifically, we found that more connected networks that allow for greater information sharing perform better when agents collaborate, but less connected networks that slow convergence work best when group members primarily copy. We also demonstrated how groups can maximize average as well as best member performance by collaborating initially and then copying later.

Our results suggest a number of directions for future research, both theoretical and empirical. For example, future models could relax the assumption that skills are randomly distributed across agents and solutions. This design decision creates a neutral baseline in our model, but implies that all skills are equally useful in expectation and that there is no correlation between pairs of problem-solving types. In practice, some specialties are more similar to one another than others. For example, a solution that is well-known to a mathematician may be more likely to also occur to a physicist than to an art historian. Some skills are also likely better suited for certain problems than others. One possible approach would be to model problem solvers as “toolboxes” comprised of multiple skills rather than having each problem solver correspond to a single skill type^8^. Overlap in skill sets could then capture correlations in problem-solving approaches. While we avoid the toolbox framework and distribute skills randomly to simplify the model and interpretation of results, future research could endow agents with multiple skills to explore how variation in group diversity impacts our findings. If agents tend to tackle problems that best match their own skill type, the benefits of collaborating with other types might be reduced.

Another model extension might be to add varying costs to copying and collaboration. Costs could be incorporated explicitly, and individual agents could then attempt to maximize the payoffs of their solutions minus costs. Alternatively, costs could be endogenized as a time penalty to communication. For example, an agent might have to give up a time step in order to communicate, or their perspective radius might be temporarily decreased. (The authors would like to thank an anonymous reviewer for this suggestion). Depending on parameters, these tradeoffs might suggest different contexts in which collaboration or copying strategies are more effective. We briefly explore one operationalization of this idea in the SI by limiting the number of contacts an individual can collaborate with in a given time step. This change to the model reduces the payoffs of high levels of collaboration and highly connected networks but does not change the main finding shown in Fig. 5 that groups do best in less connected networks when they primarily copy and better in more connected networks when they mostly collaborate.

In addition to extending the model, future work could combine modeling with behavioral experiments to test both the model assumptions and conclusions. While a number of experiments probe the relationship between networks and problem solving^11–13,29–31^, our research points to the nature of information communicated through network connections as a potential key but understudied variable in determining which networks perform best. The fact that groups often solve problems that are out of reach for their individual contributors suggests that members do more than just copy their peers, but a significant empirical question raised by our results is, in practice, when do problem solvers copy and when do they collaborate? Similarly, while a number of theoretical models point to limiting or slowing communication as a beneficial strategy^14–16,18^, most previous empirical studies find that more efficient communication leads to better solutions^11–13^, suggesting that, at least in the context of these experiments, group members tend to do more than just copy one another’s solutions. It would be informative to see if an experiment in which participants are explicitly limited to copying alone can produce outcomes in which slower communication prevails. The decision to copy or collaborate is most likely driven by incentives, so the design of reward systems to encourage dense collaboration networks that defer copying is another important next step with potential implications for science funding agencies, universities, and research and development.

Our research adds to the growing consensus that diversity benefits groups^14,16,17,31^. But when it comes to how to best structure an organization to encourage diversity, various conceptualizations of diversity operate differently. For example, Gomez and Lazer show that teams do best when individuals with similar knowledge are clustered closely together within networks, while members with different skills are dispersed more broadly^17^. Shore, Bernstein, and Lazer find that highly clustered networks promote diversity of facts that contribute to solving a problem but inhibit diversity in terms of theories that combine those puzzle pieces to suggest a solution^31^. Similarly, our results suggest that when agents collaborate, efficient networks help diffuse diverse perspectives, but when agents copy, greater network efficiency depresses solution diversity. By combining efficient networks with a collaborate first/copy later strategy, organizations can harness the exploration benefits of quickly diffusing diverse perspectives with the exploitation benefits of rapid coalescence around group leaders once progress has slowed.

## Methods

### Problem space

We generated our complex problem solution landscapes using the same procedure and parameters detailed in the article by Mason and Watts^13^. In brief, these landscapes are created by starting with a “signal” generated by a unimodal bivariate Gaussian distribution with a randomly chosen mean in the 100 x 100 problem solution grid and variance three. This signal distribution is then summed together with a sequence of pseudorandom Perlin noise distributions^32^. For robustness, we also ran simulations on a version of the NK fitness landscape as implemented by Barkoczi and Galesic^18^. Those results are described in the SI.

### Simulation procedure

Before each simulation run, we generate a new problem landscape and randomly assign each solution cell to one of *s* skill types. Each of *n* group members is also randomly assigned one of the *s* skill types and a random position on the 100 x 100 solution space. The simulation then proceeds through a sequence of discrete time steps. At each step, agents are chosen to update their position in a randomly selected order. When it is an agent’s turn to update, with probability *p* they collaborate with their neighbors and with probability 1 − *p* they copy. Agents that are selected to collaborate consider the payoffs of all of the cells adjacent to their current cell, cells within distance *r* of their current cell that match their type, and cells within distance *r* of their current cell that match the type(s) of any of their network neighbors. Agents that are selected to copy consider the payoffs of all of the cells adjacent to their current cell, cells within distance *r* of their current cell that match their type, and the cells that their network neighbors currently occupy. The agent then chooses the highest payoff solution from among these considered cells and moves to that point. If none of the payoffs of the considered cells exceeds the payoff of the agent’s current position, the agent stays where they are. Once all agents have updated, the simulation proceeds to the next time step. “The simulation terminates after the first time step in which no agent moves”. Results reported in the main text use group size *n* = 16, *s* = 100 skill types, and skill radius *r* = 6 (where the distance between cells is computed using Euclidean distance measured between the centers of the cells). Alternate parameters and specifications are reported in the SI.

## Supplementary information

Supplementary information

## Data Availability

All data presented in this study are reproducible through execution of the model source code available at this GitHub repository: https://github.com/gulsahakcakir/Group-Problem-Solving/tree/manuscript.
