# Supplementary material for: Copy or collaborate? How networks impact collective problem solving
Source: Npj Complex. 2025 Nov 27;2(1):35. doi: 10.1038/s44260-025-00058-8 (PMC12660144; doi:10.1038/s44260-025-00058-8)
Supplement: Supplementary file 1 — Supplementary information [file 44260_2025_58_MOESM1_ESM.pdf]

Supplementary Information for

Copy or Collaborate? The Interaction of Communication and Networks on Collective Problem Solving

Akçakır, Lang, and Lamberson

This PDF file includes:

Supplementary Figures S1 to S10

Supplementary Notes 1 to 6

Supplementary Figures

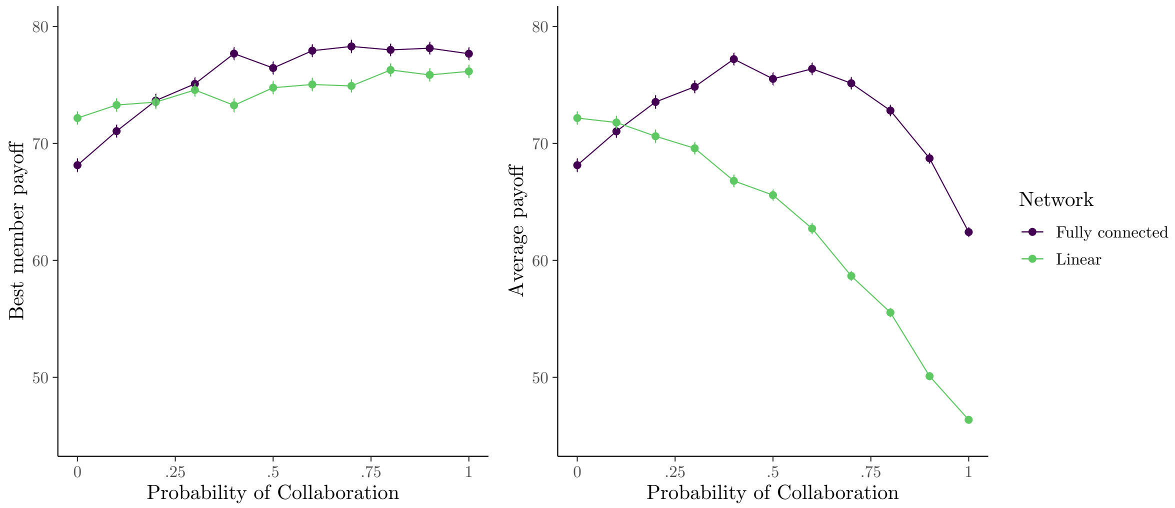

Figure S1: **Performance on the NK landscape with $N = 15$ and $K = 7$.** The maximum (left) and average (right) team payoffs in fully connected (purple) and linear (green) networks as a function of the probability of collaboration, p. Error bars are the standard errors of the mean across one thousand simulation runs.

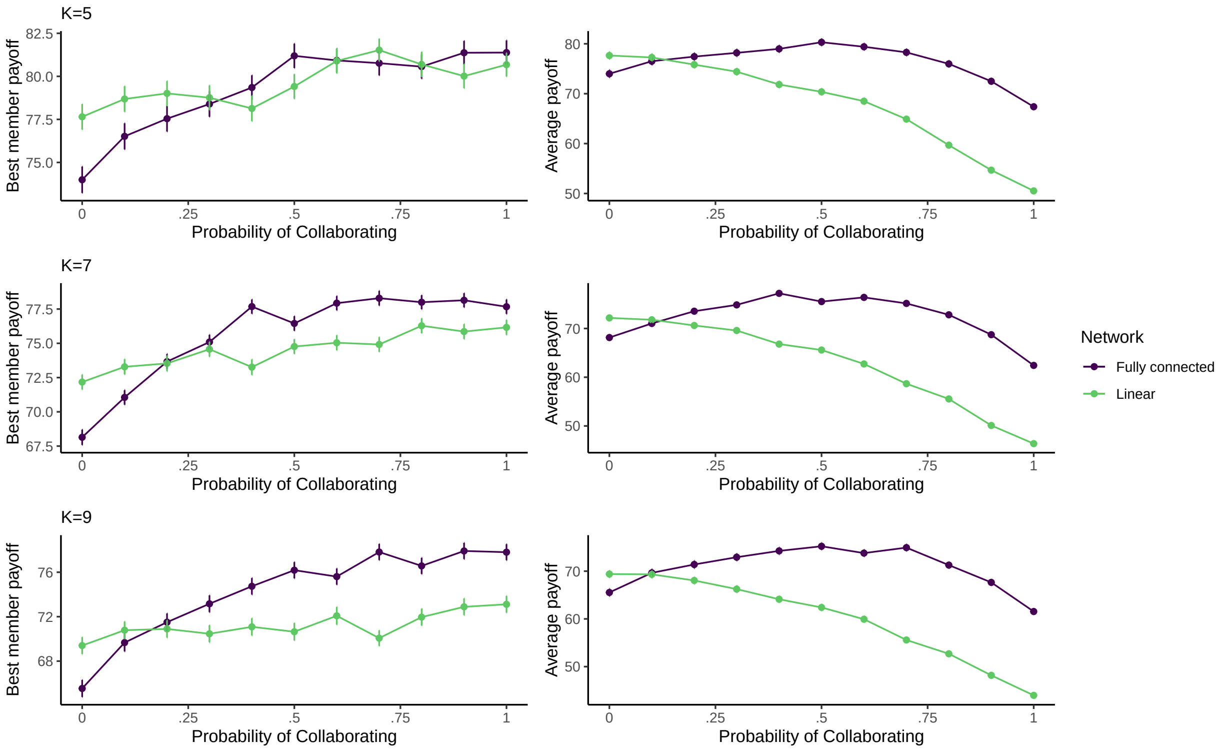

Figure S2: **Performance on the NK landscape with $N = 15$ and $K = 5, 7,$ and 9.** The maximum (left) and average (right) team payoffs in fully connected (purple) and linear (green) networks as a function of the probability of collaboration, p. Error bars are the standard errors of the mean across one thousand simulation runs.

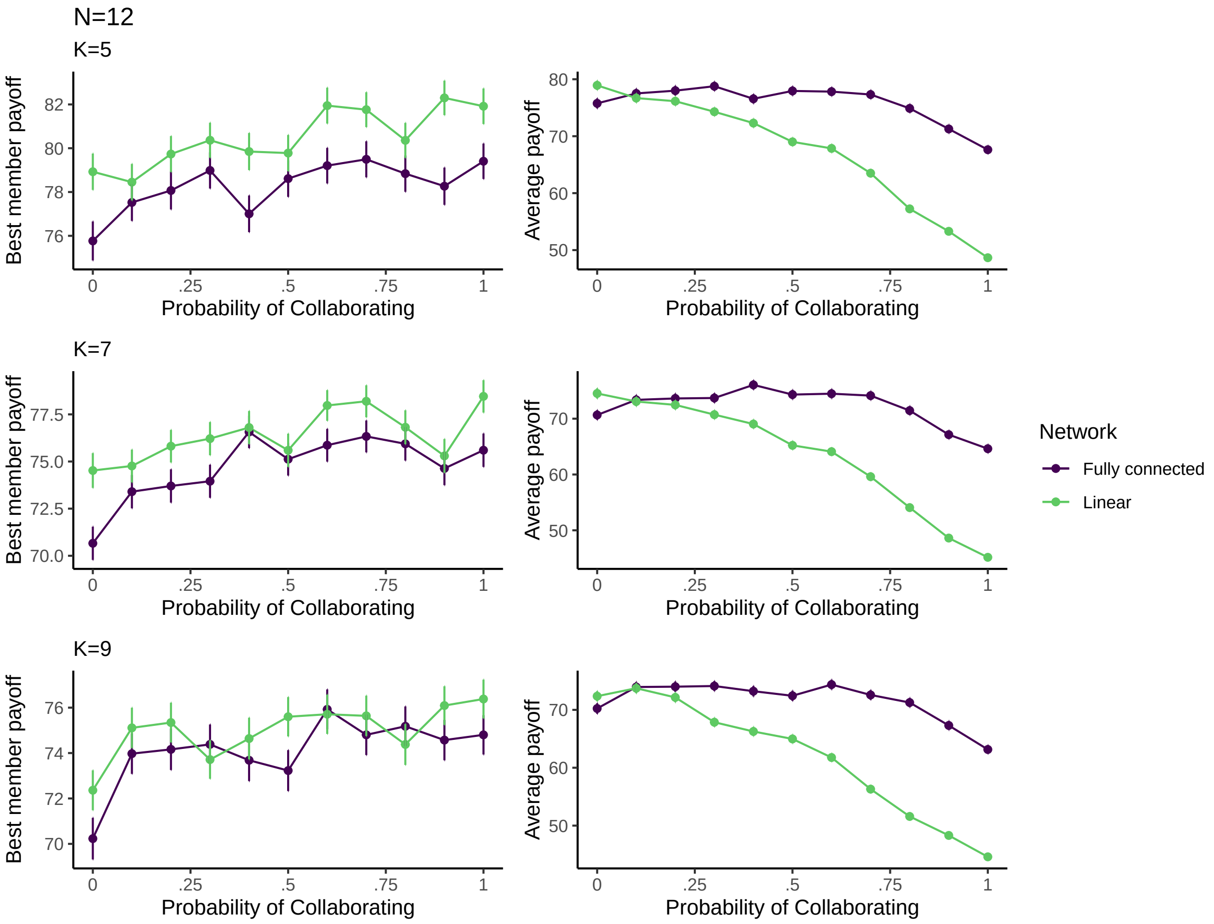

Figure S3: **Performance on the NK landscape with $N = 12$ and $K = 5, 7,$ and 9.** The maximum (left) and average (right) team payoffs in fully connected (purple) and linear (green) networks as a function of the probability of collaboration, p. Error bars are the standard errors of the mean across one thousand simulation runs.

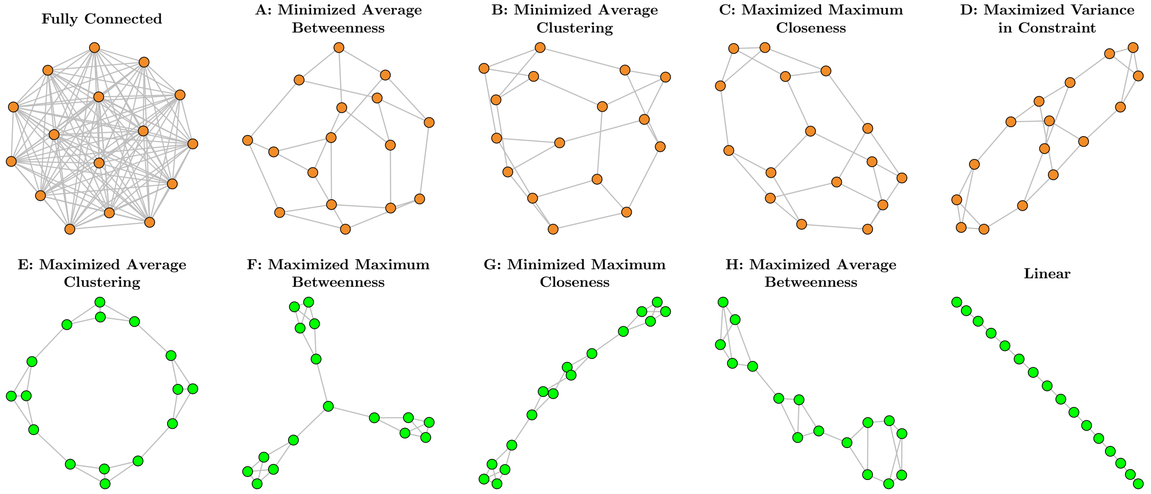

Figure S4: **Networks used in simulation experiments.** The networks are ordered and colored by efficiency, i.e., *average path length*, with “efficient” (orange) networks at the top row and “inefficient” (green) networks at the bottom row.

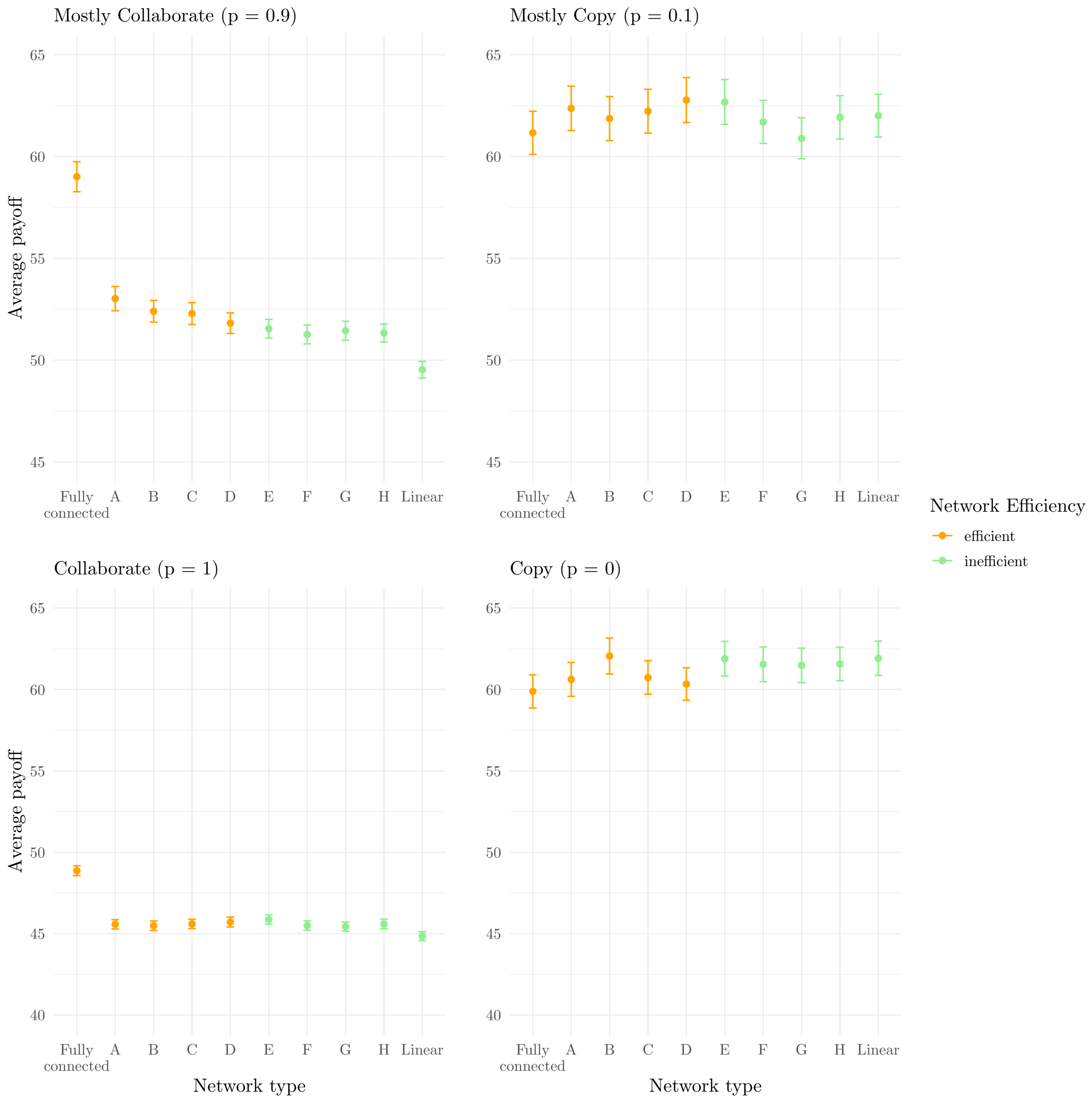

Figure S5: **Average performance on different network structures.** Average performance faceted by collaboration frequency. Networks are arranged on the horizontal axis in ascending order of average path length. Simulations were run with $n = 16$, $r = 6$, and $s = 100$ for one thousand repetitions. Error bars represent the standard errors of the mean across all repetitions.

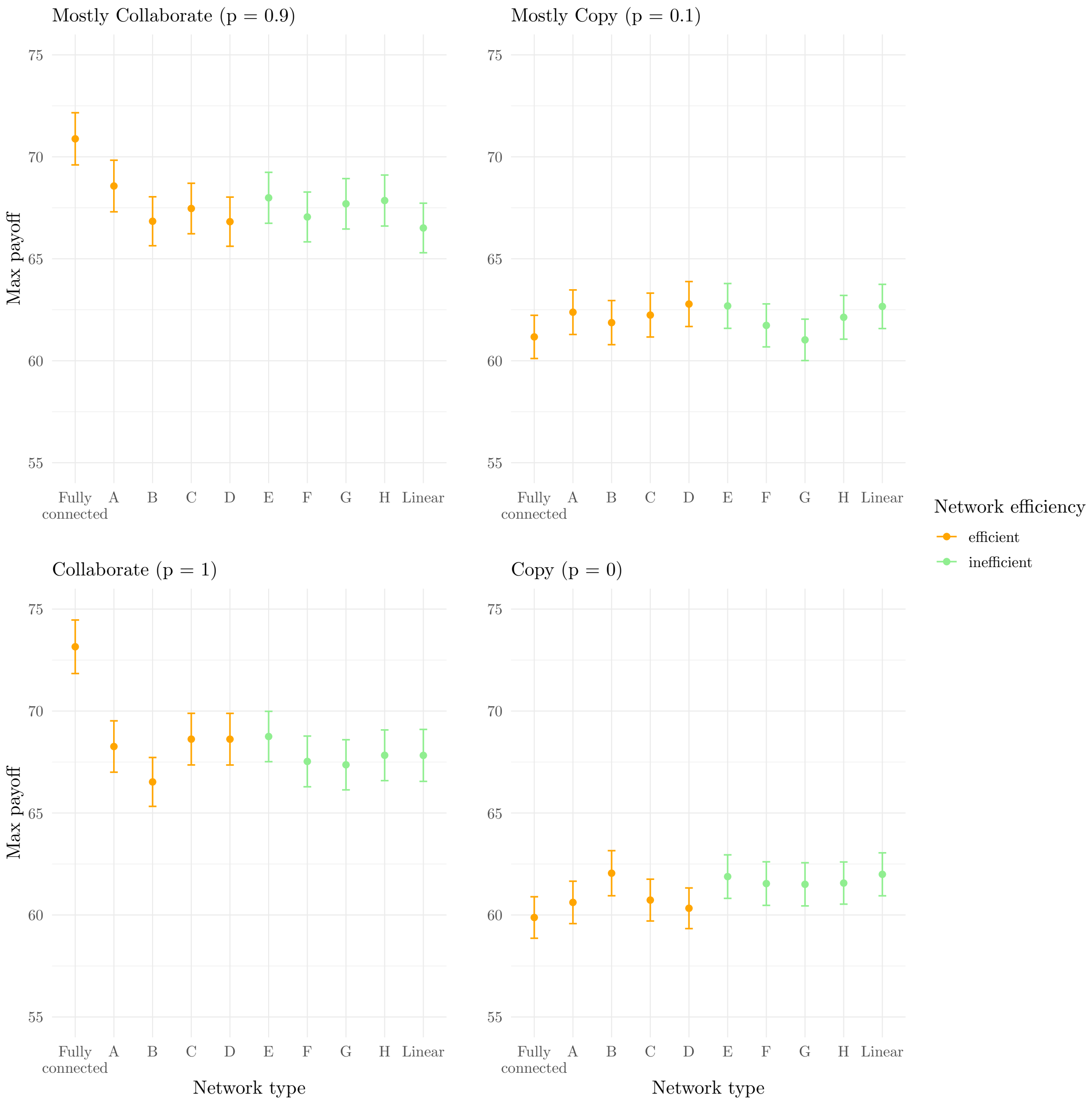

Figure S6: **Best member performance on different network structures.** Maximum performance faceted by collaboration frequency. Networks are arranged on the horizontal axis in ascending order of average path length. Simulations were run with $n = 16$, $r = 6$, and $s = 100$ for one thousand repetitions. Error bars represent the standard errors of the mean across all repetitions.

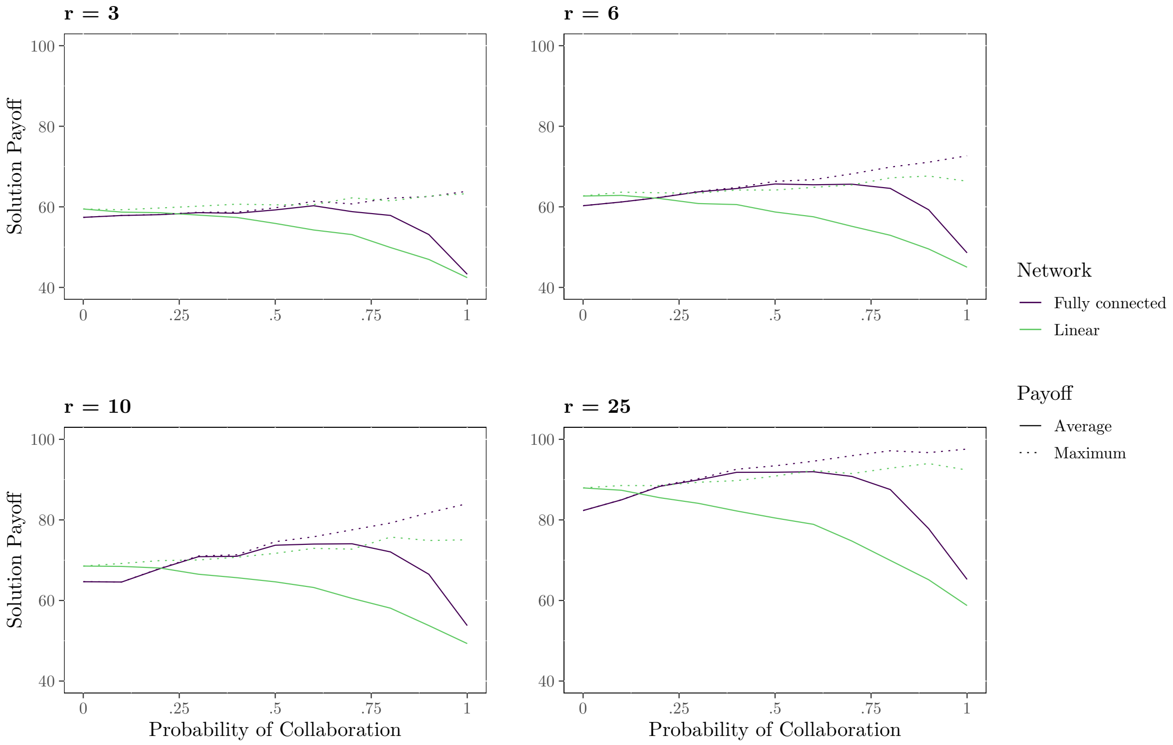

Figure S7: **Sensitivity to distance parameter.** Simulations were run for $r = \{3, 6, 10, 25\}$ with $n = 16$ and $s = 100$ held constant. Results show averages over a thousand repetitions.

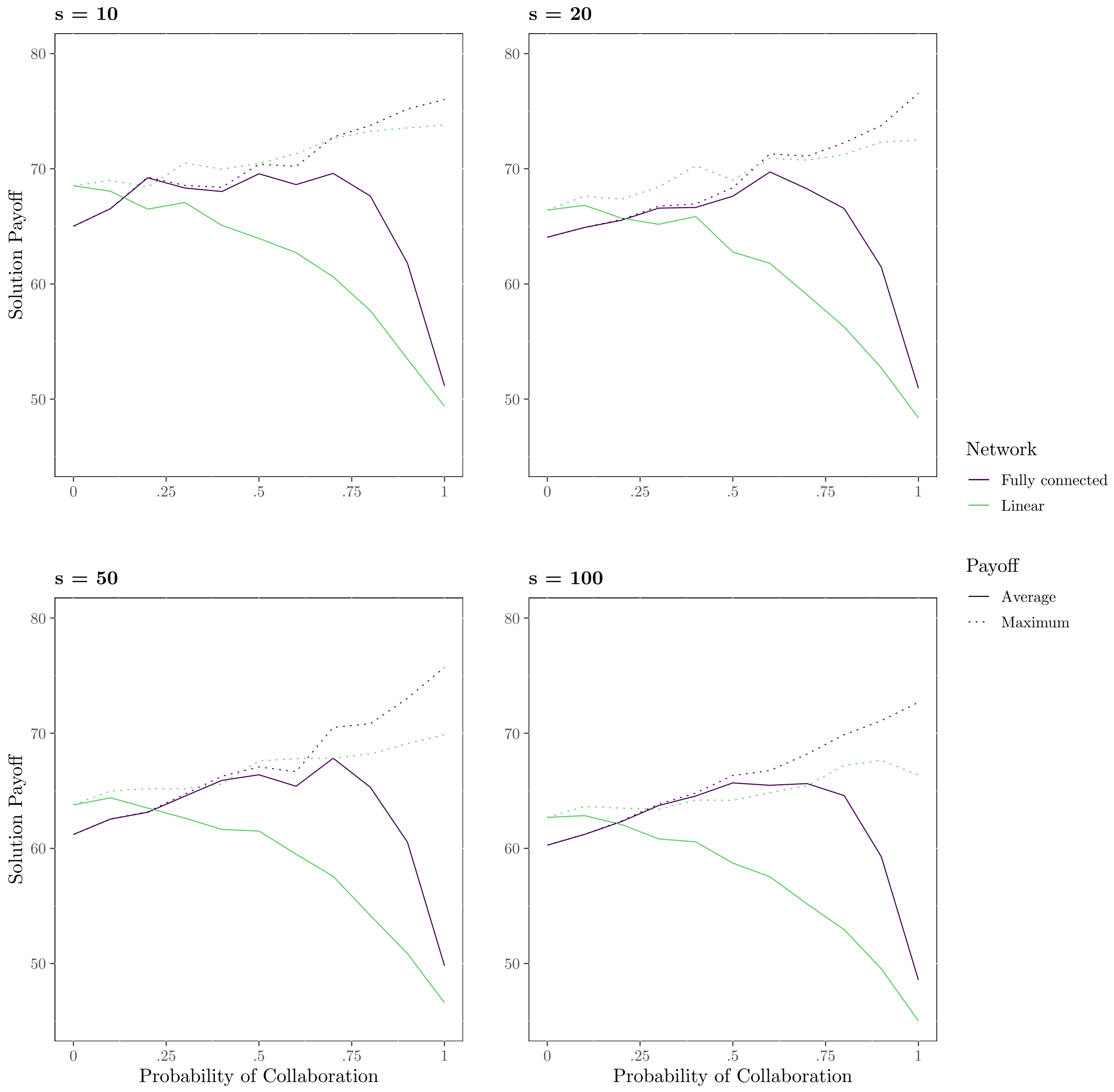

Figure S8: **Sensitivity to number of skill types.** Simulations were run for $s = \{10, 20, 50, 100\}$ with $n = 16$ and $r = 6$ held constant. Results show averages over a thousand repetitions.

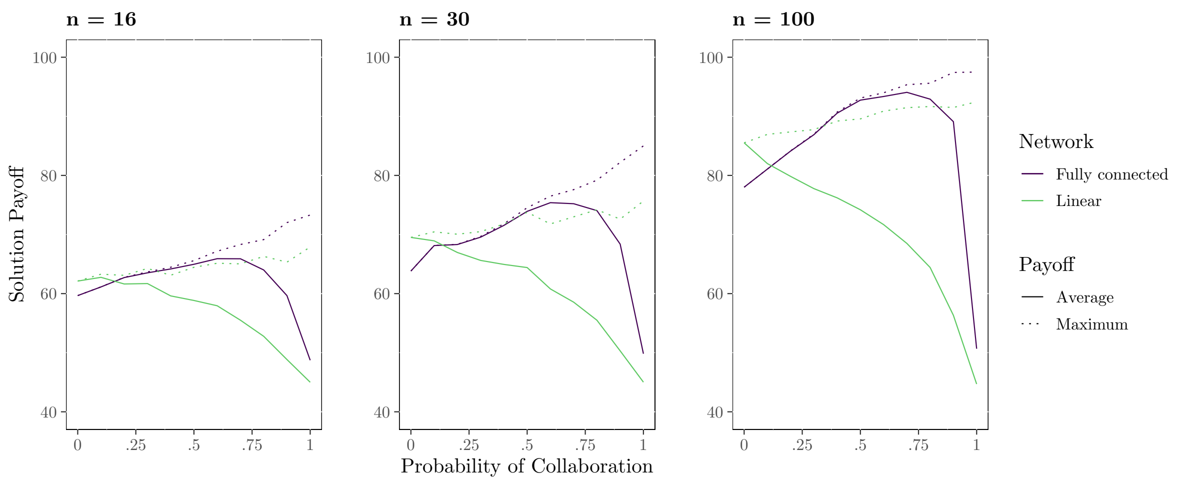

Figure S9: **Sensitivity to team size.** Simulations were run for $n = \{16, 30, 100\}$ with $r = 6$ and $s = 100$ held constant. Results show averages over a thousand repetitions.

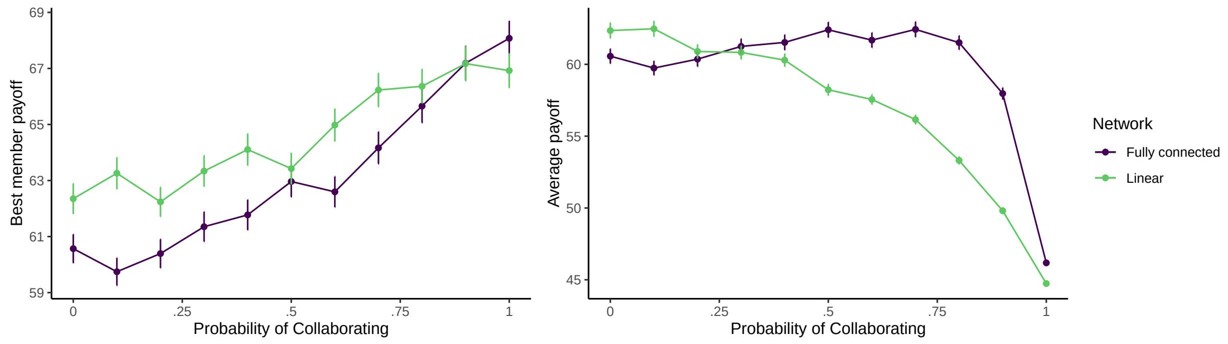

Figure S10: **Team performance when collaboration is limited to one contact per time step.** All parameters are the same as Figure 5 in the main text except that in each time step if an agent collaborates they only colaborate with one other agent chosen randomly from among their network neighbors. Results show averages over a thousand repetitions. Error bars represent the standard errors of the mean across all repetitions.

Supplementary Notes

Supplementary Note 1

Performance on the NK landscape. We test our model on NK landscapes, the dominant framework used to generate landscapes in previous studies of networked problem solving (e.g., [1, 2]). The key difference between the three-dimensional Perlin noise landscapes used in the main text and the NK landscapes in the experiments presented here is the dimensionality of the search space. Both methods generate landscapes with adjustable ruggedness, a single global optimum, and multiple local optima that effectively capture the challenges of navigating complex problem spaces. However, NK landscapes extend into multiple dimensions, introducing a greater number of interacting variables that influence fitness [3].

Supplementary Figure S1 illustrates the impact of network structure on the best member and average team performance across different collaboration probabilities on NK landscapes with $N = 15$ and $K = 7$, following [2]. We replicate our main findings that higher levels of collaboration increase best member performance but reduce average team performance. Moreover, we observe the same interaction pattern between network structure and communication strategy: the linear network is better when agents mostly rely on copying, whereas the fully connected network outperforms when collaboration is more frequent. Figures S2 and S3 illustrate the same patterns for different values of both N and k. Overall, these findings highlight that network effects are not uniform but rather moderated by the rate of collaboration among team members on a variety of complex problem solving landscapes.

Supplementary Note 2

Performance on other network structures. We extend our analysis by testing the dynamics of our model on a set of networks with distinctive properties, particularly using the networks studied in [4]. Structures of these networks are shown in Supplementary Figure S4.

Supplementary Figures S5 and S6 illustrate average team performance and best member performance, respectively, across these networks for different communication profiles. As seen in Supplementary Figure S5, teams embedded in more efficient networks tend to outperform those

in inefficient networks under high collaboration scenarios. However, when teams primarily copy, performance differences become much smaller and slightly favor networks with slower information transmission capacity. Figure S4 extends the analysis to best member performance, examining the maximum solution payoff achieved in each network. The trends largely mirror those in Figure S3, with efficient networks showing superior best member performance in high-collaboration scenarios and inefficient networks performing slightly better in low-collaboration settings.

Supplementary Note 3

Impact of distance parameter on performance. Supplementary Figure S7 depicts how decreasing agent myopia, tuned by increasing r, improves overall performance across both network structures. This trend aligns with expectations, as higher values of r enhance local search capabilities at the individual level. With a greater search radius, agents can evaluate a larger set of potential solutions at each time step, leading to more effective exploration of the landscape and, consequently, higher collective performance.

Notably, as r increases, the performance gap between the fully connected and linear networks becomes more pronounced, especially when agents collaborate with higher probability. This suggests that network efficiency amplifies the combined benefits of improved individual search and increased collaboration.

Supplementary Note 4

Impact of number of skill types on performance. Supplementary Figure S8 shows how increasing the number of skill types (s) influences team performance across different network structures and collaboration probabilities. Overall, a higher number of skill types slightly reduces performance, but the effect is minimal, and the core interaction pattern between communication form and network structure remains consistent.

As s increases, the likelihood that a fixed-size team will collectively possess a comprehensive set of skills gradually decreases. Consequently, the set of discoverable solutions on the landscape becomes more constrained, limiting overall performance. However, a higher s also distributes skill types more evenly across the solution space, potentially mitigating the disadvantages of missing

specific skills within a team. Given the relatively small effect of this parameter on performance, these dynamics seem to balance each other out.

Supplementary Note 5

Impact of team size on performance. Supplementary Figure S8 presents solution payoffs by collaboration probability and network structure, by team size (n). Larger teams tend to perform better overall, as they can more effectively divide labor and explore a broader set of potential solutions. This advantage is particularly evident in the fully connected network, where increased collaboration facilitates the dissemination of high-quality solutions, as long as teams do not overly rely on collaboration but continue to benefit from exploiting known solutions by copying.

An important trend shown in the results is that as team size increases, the gap between average and maximum performance shrinks in the fully connected network but becomes more pronounced in the linear network. This suggests that in larger teams, network efficiency plays a crucial role in bringing lower-performing members closer to top performers. These findings underscore how larger teams, when structured efficiently, can mitigate performance disparities and enhance overall problem-solving effectiveness.

Supplementary Note 6 As suggested by one anonymous reviewer, collaboration may require significantly more time than copying. As a result, individuals may be limited in the number of individuals with whom they can reasonably collaborate in a given period of time. To examine the sensitivity of our main results to this possibility, we modified the model so that whenever an agent is chosen to collaborate, rather than collaborating with all of their network neighbors, they only collaborate with a single agent chosen at random from among their network neighbors.

As the results shown in Figure S10 illustrate, this modification to the model substantially reduces the best member payoffs in the fully connected network. In the base case model, collaborating agents in the fully connected network have access to the skills of fifteen network neighbors, but with this modification that number is reduced to one. The impact on the linear network is much less because even in the base case, collaborating agents only have two neighbors, so the reduction to one is less significant. Nevertheless, even incorporating these limits on collaboration, our main finding illustrated in Figure 5 in the main text that groups do best in less connected networks when they

primarily copy and better in more connected networks when they mostly collaborate continues to hold.

Supplementary References

[1] Lazer, D. & Friedman, A. The network structure of exploration and exploitation. *Administrative Science Quarterly* **52**, 667–694 (2007).

[2] Barkoczi, D. & Galesic, M. Social learning strategies modify the effect of network structure on group performance. *Nature Communications* **7**, 13109 (2016).

[3] Kauffman, S. A. & Levin, S. Towards a general theory of adaptive walks on rugged landscapes. *Journal of Theoretical Biology* **128**, 11–45 (1987).

[4] Mason, W. & Watts, D. J. Collaborative learning in networks. *Proceedings of the National Academy of Sciences* **109**, 764–769 (2012).
